# Window Size Impact in Human Activity Recognition

**DOI:** 10.3390/s140406474

**Published:** 2014-04-09

**Authors:** Oresti Banos, Juan-Manuel Galvez, Miguel Damas, Hector Pomares, Ignacio Rojas

**Affiliations:** Department of Computer Architecture and Computer Technology, Research Center for Information and Communications Technologies—University of Granada (CITIC-UGR), C/Calle Periodista Rafael Gomez Montero 2, Granada E18071, Spain; E-Mails: jonas@correo.ugr.es (J.-M.G.); mdamas@ugr.es (M.D.); hector@ugr.es (H.P.); irojas@ugr.es (I.R.)

**Keywords:** activity recognition, segmentation, windowing, window size, wearable sensors, inertial sensing, human behavior inference

## Abstract

Signal segmentation is a crucial stage in the activity recognition process; however, this has been rarely and vaguely characterized so far. Windowing approaches are normally used for segmentation, but no clear consensus exists on which window size should be preferably employed. In fact, most designs normally rely on figures used in previous works, but with no strict studies that support them. Intuitively, decreasing the window size allows for a faster activity detection, as well as reduced resources and energy needs. On the contrary, large data windows are normally considered for the recognition of complex activities. In this work, we present an extensive study to fairly characterize the windowing procedure, to determine its impact within the activity recognition process and to help clarify some of the habitual assumptions made during the recognition system design. To that end, some of the most widely used activity recognition procedures are evaluated for a wide range of window sizes and activities. From the evaluation, the interval 1–2 s proves to provide the best trade-off between recognition speed and accuracy. The study, specifically intended for on-body activity recognition systems, further provides designers with a set of guidelines devised to facilitate the system definition and configuration according to the particular application requirements and target activities.

## 1. Introduction

During the last few years, a tremendous interest in the evaluation of people's habits and daily routines has awakened. The analysis of human behavior has been demonstrated to be of key value to better understand people's necessities and demands. This understanding is of utility in a wide variety of fields, from education, medicine or sociology, to gaming or other kinds of industries with a demonstrated potential impact on society [1]. Nevertheless, healthcare, assistance and wellness are possibly the fields that most actively leverage the knowledge gained from the analysis of human behavior. Here, the use of this information is, for example, devised for people's health empowerment. Promoting healthier lifestyles (e.g., encouraging exercising [2,3]), preventing unhealthy habits (e.g., tobacco use or unwholesome food [4,5]), detecting anomalous behaviors (e.g., fall detection [6–8]) or tracking conditions (e.g., mobility worsening due to aging or illnesses [9]) are different applications which may profit from the inference of human behavior.

The inference of human behavior could be performed in different ways; however, a mainstream discipline stands out among the others. Also known as activity recognition, it aims at interpreting people's movements, actions and goals through the use of diverse sensing technologies. Activity recognition normally makes use of sensors on and around the subject to register their movements, while expert systems employ the monitored data to detect the performed activities. Among the diverse technologies used for activity detection, on-body sensing proves to be the most prevalent monitoring technology.

On-body or wearable activity recognition systems normally consist of a set of sensors attached to the person's body that deliver signals (data streams) of diverse modalities. These could be of a continuous or discrete nature, but in any case, the data stream must be segmented in data windows for processing. The segmentation process should be normally defined depending on the particular requirements of the application for which the recognition system is devised. Some systems are planned for detecting a specific activity; thus, a particular segmentation could be found to optimize the recognition quality. Other applications may need to identify several activities or actions, therefore requiring a data partitioning that works well on average for the target activities. Moreover, depending on the addressed problem, a fast identification may be needed (e.g., fall detection) or, conversely, it may not have special time requirements (e.g., kilometers walked in a day). Since reducing the recognition time (*i.e.*, segmentation) may have an influence on the system performance, a tradeoff between detection time and accuracy should be considered by recognition system designers. Despite the importance of this, little work has been devoted to investigating this fact.

In this work, we present an extensive study of the effects of segmentation for diverse recognition techniques and activities. Considered the sliding window approach, the most widely used segmentation method, we evaluate the performance of several recognition systems for an extensive set of window sizes that also covers the values used in previous works. This characterization is defined for a wide variety of representative activities. The rest of the paper is structured as follows. In Section 2, an extensive review of the activity recognition segmentation process is presented. Section 3 briefly describes the activity recognition methodology used in this study. Next, the results obtained for the different experiments performed are presented. These results are subsequently discussed in Section 5, while our final conclusions are summarized in Section 6.

## 2. State of the Art

Segmentation corresponds to the process of dividing sensor signals into smaller data segments. This process has been performed in different ways in the activity recognition field. Most of the segmentation techniques could be categorized into three groups, namely activity-defined windows, event-defined windows and sliding windows. The main contributions to each category for on-body sensing activity recognition are summarized in Table 1.

The activity-defined windowing procedure consists of a partitioning of the sensor data stream based on the detection of activity changes. Initial and end points are determined for each activity, prior to explicitly identifying the specific activities. In the literature, diverse methods have been proposed to identify activity-transition points. For example, changes between activities could be identified through the analysis of variations in the frequency characteristics. In this regard, Sekine *et al.* [10] proposed a model based on wavelet decomposition to detect frequency changes for three walking activities (level walking, walking upstairs and walking downstairs) from a continuous record. A similar approach is used in [11], though only a subset of the activity window is eventually used for classification. In order to improve the activity change detection, Yoshizawa *et al.* [12] proposed a heuristic method that differentiates among static and dynamic actions. The identification of initial and end points could be also approached by leveraging the user feedback. This way, in [13,14], the monitored volunteers are made to participate in the segmentation process by requiring them to set initial and end points in a handheld device. Less obtrusively, in [15], the subjects are asked to stand still for a few seconds to better identify the start and stop of each activity. Activity windows are also defined in an offline manner for activities of a long duration. In [16], this approach is applied to activities, such as walking, running and jumping, which are partitioned into 1-min duration segments. Although these could be strictly considered activity-defined approaches, they are rarely devised for recognition purposes, but better planned for labeling procedures.

Some activities could be better recognized as a sequence of movements or actions performed in a certain order. This is the case of sporadic activities, such as household activities (e.g., meal preparation, room cleaning), in which the activity or gesture occurs sporadically and is interspersed with other activities or gestures. For gesture recognition or isolated movement detection, the identification of specific events is particularly advised. The event-defined approach consists of locating specific events, which are further used to define successive data partitioning. Since the events may not be uniformly distributed in time, the size of the corresponding windows is not fixed. Gait analysis has principally benefited from this type of analysis. Concretely, the detection of heel strikes (the initial floor contact) and toe-offs (the end of floor contact) events is normally pursued here. In [17,18], the detection of the initial and the end contact of the foot with the ground is performed through analyzing the foot's linear acceleration. Foot [19] and shank [20] sagittal angular velocity is also utilized to identify these events. Benocci *et al.* [21] recognizes walking by using a model that identifies the gait cycle on a single foot tagged through a heel strike event. Sant'Anna and Wickström [22] presents a symbol-based method used to detect the phases of the gait. Interpreting the acceleration signal, heel strikes are reflected as a valley and large variances, whilst toe-offs are presented as a peak. More recently, Aung *et al.* [23] proposed the use of a simple Gaussian mixture model to classify data samples into heel strike, toe-off or no event categories. As for the activity-defined approach, the events could be also identified through external mechanisms. The registration of these events could be, for example, performed through a stopwatch. In [24], the stopwatch count is started when the hind foot first crossed a given start line and then stopped when the lead foot first crossed the end line. Again, this kind of approach is restricted to laboratory settings or recognition under expert supervision, which is found to be of little use in real settings. Both activity-defined and event-defined methods are particularly interesting for spotting purposes; however, the size of the window normally determines that a subsegmentation process is required.

**Figure 1.**
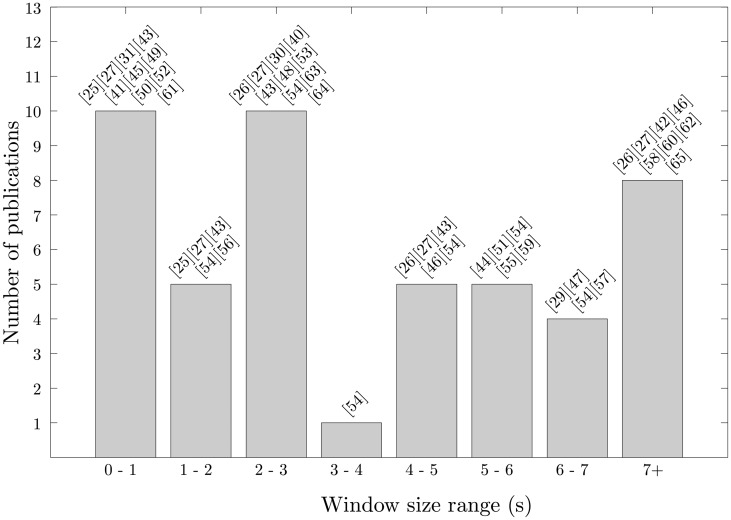
Distribution of the activity recognition research studies presented in Tables 2 and 3 based on the window size.

The sliding window approach, hereafter referred to as “windowing”, is the most widely employed segmentation technique in activity recognition. Its implementational simplicity and lack of preprocessing determines the windowing approach as ideally suited to real-time applications. Here, the signals are split into windows of a fixed size and with no inter-window gaps. An overlap between adjacent windows is tolerated for certain applications; however, this is less frequently used. A range of window sizes have been used in previous studies (Figure 1) from 0.1 s [25] to 12.8 s [26] or more [27,28], with some studies including a degree of overlap between windows [29–31]. Tables 2 and 3 present an extensive review for the windowing approach. The sliding window approach has been proven to be especially beneficial for the recognition of periodic (e.g., walking, running) and static activities (e.g., standing, sitting) and of questionable utility for the detection of sporadic activities. As has been mentioned, sporadic activities require a more sophisticated segmentation process given their complex and interspersed nature.

## 3. Activity Recognition Methods

**Figure 2.**
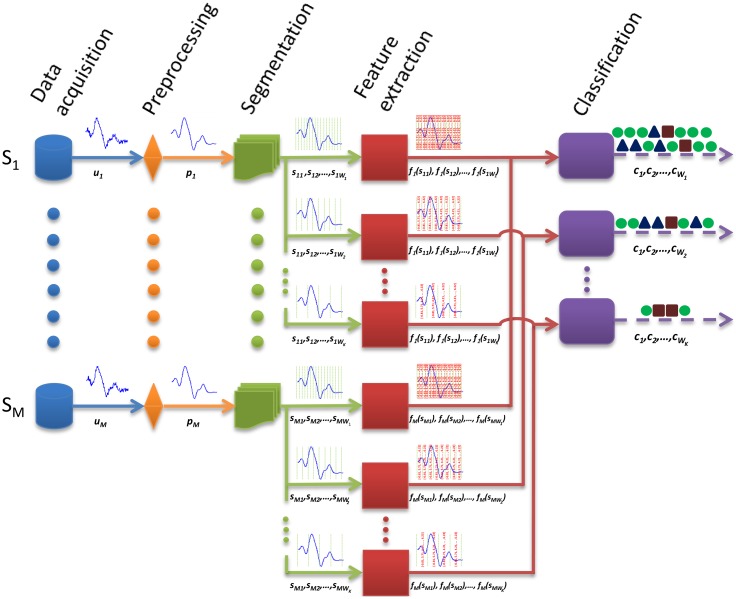
Different stages of the activity recognition chain (ARC). An example of the correlation of the windowing approach and subsequent levels of the ARC is shown. Here, different window sizes are depicted particularly. Concretely, *M* sensors deliver raw signals (*u*_1_, *u*_2_, …, *u*_M_), which are subsequently processed (*p*_1_, *p*_2_, …, *p_M_*). The signals are partitioned into data windows of size *W_k_* (e.g., s_1_*_W_k__*, s_2_*_W_k__*, …, s*_MW_k__*). For each window, *k*, a set of features are extracted and aggregated in a single feature vector (*f*_1_(*s*_1_*_W_k__*), *f_2_*(*s*_2_*_W_k__*), …, *f_M_*(*s_MW_k__*)) that is used as the input to a classifier. The classifier yields a class (*c_W_k__*) that represents the identified activity.

Signal segmentation is one of the stages of the activity recognition process, also known as the activity recognition chain (Figure 2). Concretely, a set of nodes (sensors) usually delivers a stream of raw unprocessed signals, which represent the magnitude measured (e.g., acceleration). The registered information may be disturbed by electronic noise or other kinds of artifacts. These disturbances are sometimes removed through a filtering process [18,66]; however, this is not always applied, since it may imply a certain information loss. In order to capture the dynamics of the signals, these are partitioned into segments of data. As already described in Section 2, different techniques could be used for this purpose, albeit the windowing approach is the most widely used for its simplicity and tractability. In Figure 2, diverse windowing procedures, respectively corresponding to different window sizes, are depicted. Subsequently, a feature extraction process is carried out to provide a handler representation of the signals for the pattern recognition stage. A wide range of heuristics [67], time/frequency domain [44,45] and other sophisticated mathematical and statistic functions [68] are commonly used. The feature vector is provided as the input of the classifier or reasoner [69], ultimately yielding the recognized activity or class to one of those considered for the target problem.

As can be seen from Figure 2, a feature vector is computed for each data window, thus also determining the rate at which the classification or recognition is performed. Therefore, reducing the window size translates into a faster detection at the expense of using less data for the feature computation. The tradeoff between window size and recognition performance is extensively analyzed and characterized for diverse activity recognition models in this work.

## 4. Results

### 4.1. Experimental Setup

To evaluate the impact of signal segmentation on the recognition process, an adequate representative dataset must be used. Taking into account the characteristics of the sliding window technique and its normal use (Section 2), the activities of a periodic and static nature are particularly considered. Here, one of the most complete activity recognition benchmark datasets is used [70]. This dataset comprises motion data recorded from 17 volunteers of diverse profiles performing 33 fitness activities (Table 4) while wearing a set of nine inertial sensors attached to different parts of their bodies. This dataset not only stands out for the number of considered activities, but for the diversity of body parts involved in each one (e.g., lateral elevation of the arms *vs*. knees bending), the intensity of the actions (e.g., cycling *vs*. waist rotation) and their execution speed or dynamicity (e.g., running *vs*. standing while hand-clapping). The activities are collected in an out-of-lab environment with no constraints on the way these must be executed, with the exception that the subject should try their best when executing them. The use of multiple sensors also permits measuring the motion (namely, the acceleration, the rate of turn and the magnetic field orientation) experienced by each body limb and trunk, thus better capturing the body dynamics. Here, only the acceleration data is considered for the study, since this proves to be the most prevalent sensor modality in previous activity recognition contributions [71,72]. The dataset provides data for three different scenarios, one for a default setting and two others for the study of sensor anomalies (out of the scope of this work); thus, only the data for the default setup is here used.

The implemented recognition methods (see Section 3) are now described. No preprocessing of the data is applied to avoid the removal of relevant information. This is normal practice when the activities are diverse, even more when the quality of the registered data permits it. The segmentation process basically consists of a non-overlapping sliding window approach. Different window sizes are used for evaluation, concretely ranging from 0.25 s to 7 s in steps of 0.25 s. This interval comprises most of the values used in previous activity recognition systems. The segmentation process is applied for each activity in isolation. Three feature sets (FS) are respectively used for evaluation: FS1 = “mean”, FS2 = “mean and standard deviation” and FS3 = “mean, standard deviation, maximum, minimum and mean crossing rate”. These are some of the features most widely used in activity recognition [16,29,41,44,58] for their discrimination potential and ease of interpretation in the acceleration domain. Likewise, four of the most extensively and successfully machine learning techniques used in previous activity recognition problems are considered for classification: C4.5 decision trees (DT, [73]), k-nearest neighbors (kNN, [74]), naive Bayes (NB, [75]) and nearest centroid classifier (NCC, [76]). The k-value for the KNN model is empirically set to three.

System evaluation is carried out through a cross-validation process. Although leave-one-subject-out cross validation (LOOXV) has been used in the literature, here, a ten-fold cross-validation (10-fold XV) process is rather chosen to compare the diverse models. In fact, as summarized in [77] and according to [78,79], LOOXV is the best technique for risk estimation, whereas 10-fold XV is the most accurate approach for model selection. Moreover, this process is repeated 100 times to ensure statistical robustness, as well as to procure an asymptotic convergence to a correct estimation of the system performance [80].

The *F*_1_-*score* [81], a combination of precision and recall measures, is used as a performance metric to assess the quality of the recognition for each system design. This metric is particularly interesting for its robustness to class imbalance, which happens to occur when there are more instances for some activities than for others. The *F*_1_-*score* ranges between [0,1], where one represents an optimal recognition capabilities, whilst zero corresponds to a system that is not capable of recognition at all.

### 4.2. Global Evaluation

**Figure 3.**
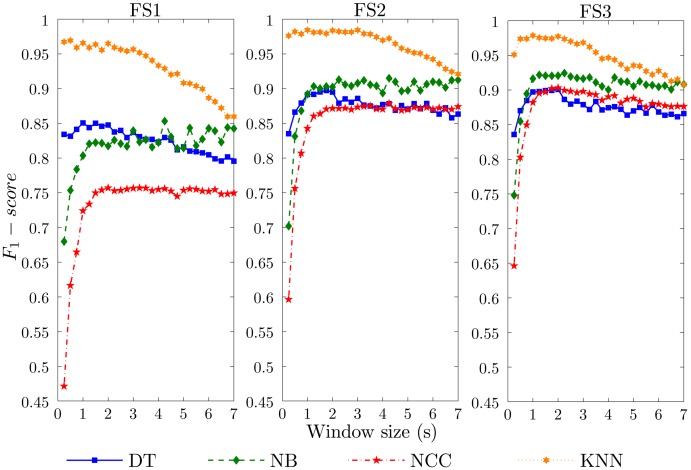
Effect of the data window size on the activity recognition system performance (*F*_1_-*score*). Twelve recognition systems, respectively, corresponding to the combination of three feature sets (FS1, FS2, FS3) and four classification models (DT, NB, NCC, KNN) are evaluated.

In this section, we analyze the general effects of the windowing operation on the activity recognition process. The performance results for diverse window sizes and each specific methodology are depicted in Figure 3. At first glance, the performance tendency for each individual classification technique is maintained for all feature sets. This determines that these results could be, in principle, generalized to other recognition models of a similar nature. Systems based on FS3 (the richest feature set considered) provide better performance than for FS2, which, in turn, notably improves the results obtained for FS1. This difference among the results for FS1, FS2 and FS3 may indicate that the use of more features may lead to improved results. Thus, the reported results could be considered a lower bound on the recognition performance.

The classification paradigm determines the impact of the window size on the recognition performance. The NB and NCC models show an increasing performance as the size of the window grows. The minimum performance is obtained for 0.25 s, which nevertheless increases up to 30% when the window is enlarged to 1 s. Actually, a “cut-off” window size is found at 1 s for all feature sets. From that value on, no significant benefits are obtained in general. For NB-FS1, less than a 5% improvement is achieved for some random window sizes when compared to the performance at 1 s. This also applies to a lesser extent for the NB-FS2 model. Conversely, increasing the window size more than 2 s entails a worsening of the recognition performance for NCC-FS3. DT shows a top performance for window sizes between one and 2 s. Upper and lower values to these generally decrease the performance of the recognizer. The KNN model stands out among all evaluated techniques and allows us to maximally reduce the window size. This technique provides the highest performance, with an *F*_1_-*score* above 0.95 for the simplest realization (FS1) and close to one for FS2 and FS3, all for minimum window sizes (0.25 s–0.5 s). For window sizes higher than 2 s for FS1 and FS3, and 3 s for FS2, the performance of the KNN systems decreases monotonically. The lowest performance is achieved for a window size of 7 s, which, for some cases, is up to 15% less than the baseline.

### 4.3. Activity-Specific Analysis

A global evaluation is of utility to have an overall view of the segmentation effects on the recognition process. Nevertheless, it is also found to be of interest to particularize this study to each specific considered activity. Thus, in the following, an extensive analysis of the systems recognition capabilities for the target activities is presented.

**Figure 4.**
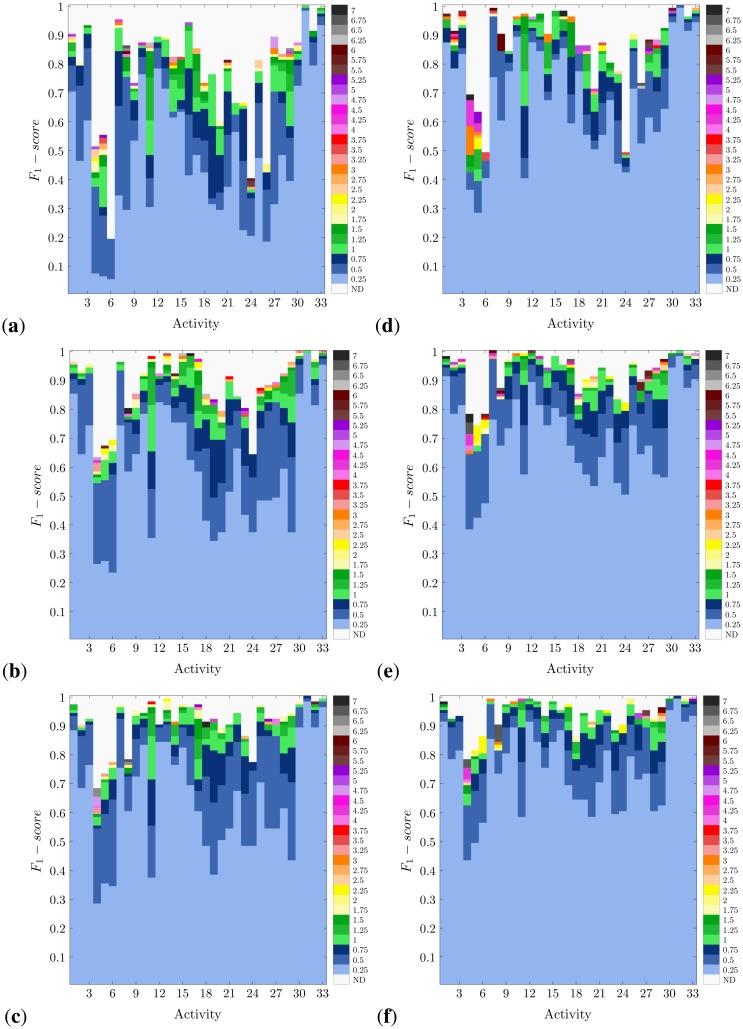
Activity-specific recognition performance for diverse window sizes and methodologies (<classification paradigm>-<feature set>): (**a**) NCC-FS1; (**b**) NCC-FS2; (**c**) NCC-FS3; (**d**) NB-FS1; (**e**) NB-FS2; and (**f**) NB-FS3. The minimum window size required to achieve a specific *F*_1_-*score* is depicted. No color is specified (not defined, ND) for performance values that may not be achieved for any of the window sizes and methodologies.

**Figure 5.**
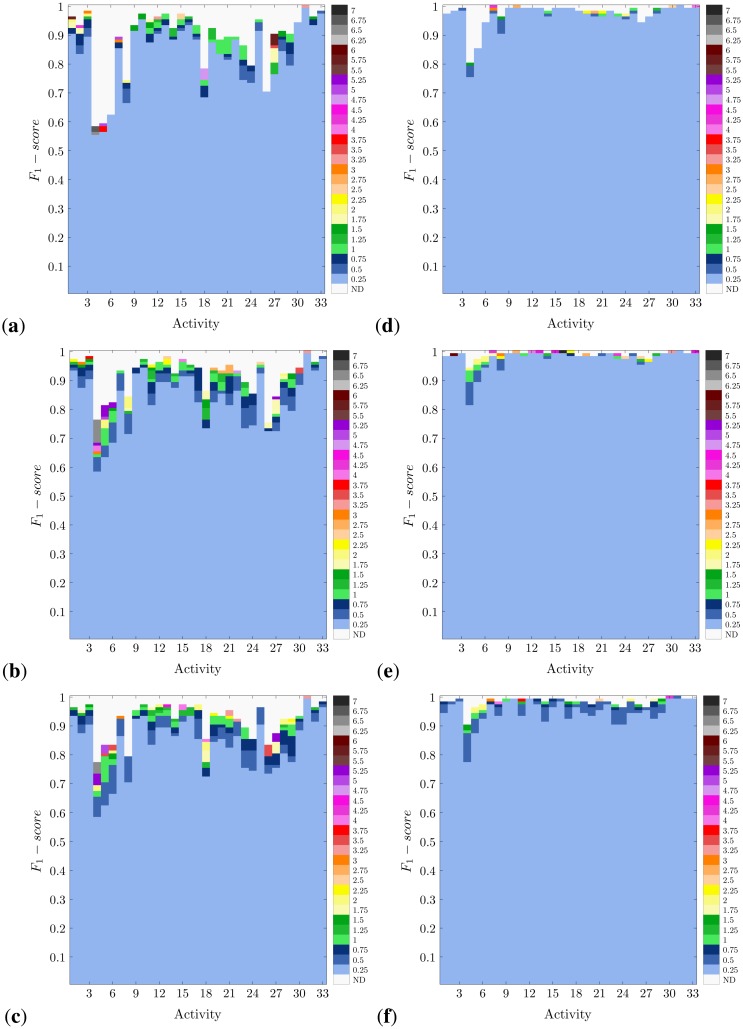
Activity-specific recognition performance for diverse window sizes and methodologies (<classification paradigm>-<feature set>): (**a**) DT-FS1; (**b**) DT-FS2; (**c**) DT-FS3; (**d**) KNN-FS1; (**e**) KNN-FS2; and (**f**) KNN-FS3. The minimum window size required to achieve a specific *F*_1_-*score* is depicted. No color is specified (not defined, ND) for performance values that may not be achieved for any of the window sizes and methodologies.

In Figures 4 and 5, the activity-specific recognition performance achieved for each methodology and for diverse window sizes is presented. Actually, not all window sizes are highlighted, but the minimum values that are necessary to obtain a certain performance. This comes from the idea of reducing the window size as much as possible, which corresponds to one of the normal design criteria. The other more habitually sought criterion is to maximize the recognition confidence. This way, these figures are devised as a perfect means to visually inspect the trade-off between performance and window size for each specific activity.

As expected from the results shown in Figure 3, the richer the feature set used, the higher the recognition performance obtained. Likewise, the best performance is observed for the systems based on DT and, foremost, KNN. Not only is KNN the most accurate method, but the one that maximally minimizes the required window size. In either case, the demonstrated recognition capabilities of all these systems apply differently to each activity type. Thus, for example, 4–6 prove to be the most difficult activities to be recognized. This happens to occur for all methodologies, although to a much lesser extent for KNN. The worst results are obtained for the NCC-FS1 approach, for which Activity 6 records a *F*_1_-*score* of 0.2. Activities 4–6 correspond to very short actions, concretely various types of jumps. Therefore, the difficulty when detecting these activities could derive from the small amount of information registered during the execution of an instance of these actions and their similarity. Including more data (*i.e.*, increasing the window size) serves to improve the recognition performance (up to 40% for NB and NCC), yet this is insufficient for practical use. Conversely, KNN perfectly copes with the challenge of distinguishing among these three activities and even for very reduced window sizes. KNN has proved in previous works to operate well for gesture recognition [82,83], which here supports the learning of subtle differences among activities of that short duration. Other activities, such as 24 (shoulders low-amplitude rotation) and 26 (*knees alternating to the breast*), are also hardly recognizable for NB, NCC and DT with FS1; however, this is enhanced when a richer feature set is used.

Activities that involve movements of the complete body are more easily recognized. Thus, for example, different types of translation (e.g., Activities 1–3, walking and running) or sports exercises (Activities 31–33, rowing, elliptical bike and cycling) are accurately recognized (*F*_1_-*score* > 0.9) for all methodologies and almost absolutely for KNN. Even when good results are obtained for the simplest realization (FS1), the use of more informative feature sets makes it possible to significantly reduce the size of the windows (*i.e.*, from windows of 6 s or more to windows of 1 s or less). Furthermore, in this line, the activities that involve specific trunk movements are fairly detected. Trunk twists, waist rotations and lateral bends are examples of these activities (Exercises 9–17). The reason why all these activities are better identified is possibly a consequence of having informative data coming from several body parts. Not only is this important for the sake of recognition, but for reducing the window size requirements. Since these activities involve the movement of most body parts, the data captured from these better describe the performed action. Thus, less data are, in principle, required for the activity detection; otherwise, the window size may be reduced. On the contrary, when some body parts do not experience a relevant movement or are similarly displaced for a set of actions, the information monitored on these parts becomes of little utility for discrimination.

It is also worth noting that for some activities, the required window size could be significantly reduced just by relaxing the performance conditions. Thus, for example, Activity 16 (lateral bend with arm up) may be recognized through the KNN-FS2 model with a maximum level of confidence (*F*_1_-*score* = 1) when a 7 s window is used. By tolerating a recognition performance of 0.99 (*i.e.*, a 1% drop), the window size could be reduced to just 0.25 s. Something similar could be seen for DT and Activity 31 (rowing), allowing for a shortening from 3.25 to 0.25 s at the expense of a subtle performance drop.

**Figure 6.**
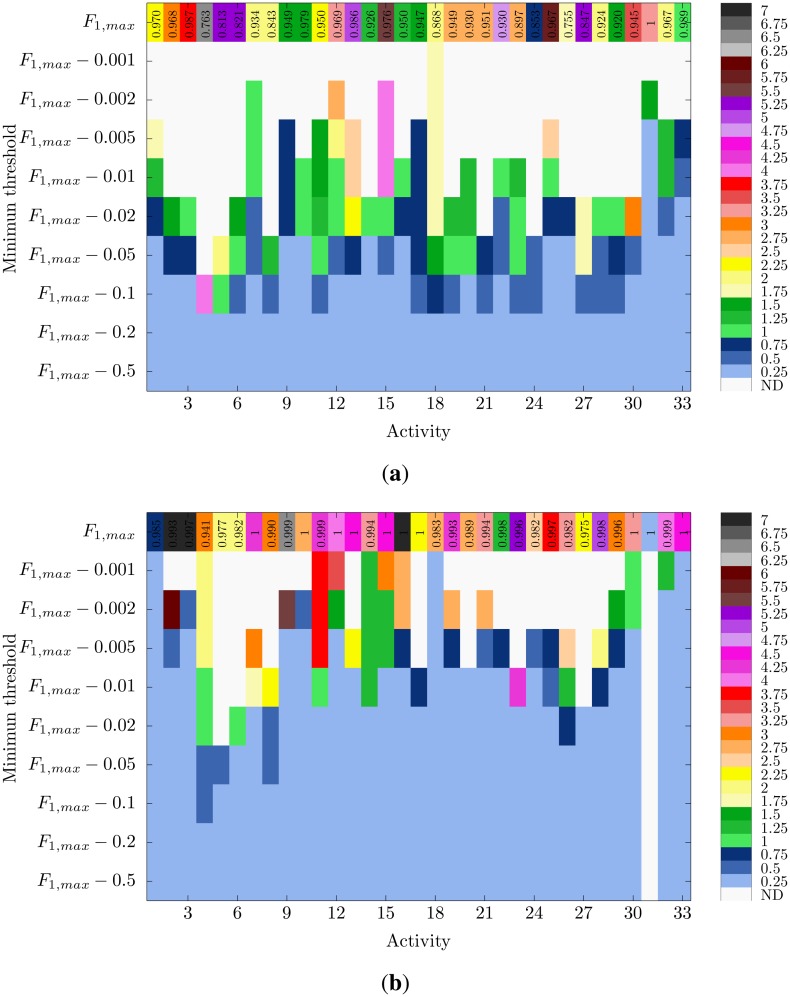
Minimum window size required for diverse performance thresholds. The threshold values are respectively calculated from the maximum *F*_1_ – *score* that could be achieved for the recognition of each activity (represented on top). The results for two particular recognition methodologies are shown: (**a**) DT-FS2; and (**b**) KNN-FS2. Non-colored spots (not defined, ND) correspond to performance values for which no window enhancement may be obtained.

The optimization of the window size could be better seen in Figure 6. Here, two examples of the trade-off between performance and window size, respectively, applying to the DT-FS2 and KNN-FS2 methods are presented. Now, for the particular case of DT and Activity 31 (Figure 6a), a 0.002 reduction of the maximum performance already allows us to narrow down the window size to 1.5 s. If the reduction is of 0.005, the minimum window size is applicable. The detection of other activities, such as 15 (trunk lateral bend) and 25 (arm inner rotation) may be also sped up from 5.5 s to 0.25 s by reducing the top performance in 5%, yet keeping a *F*_1_-*score* of more than 0.92. For KNN-FS2, the results are even more promising. A penalty of 2% with respect to the maximum performance (here, close to one for almost all activities) allow us to use the minimum window size for 28 out of the 33 activities. This performance drop translates into *F*_1_-*score* values of 0.92 at worst. Nine of these 28 (concretely, Activities 7, 10, 12, 13, 15, 16, 17, 30 and 33) are recognized with a confidence level of 0.98. Again, these are activities that involve movements of the complete body. Other examples for which an outstanding window size reduction could be applied at the expense of a negligible performance drop (0.001) could be seen for Activities 16, 18 and 33. This further applies to Activity 1 (walking), which could be detected with a confidence of 0.984 for a 0.25 s window size. This result is encountered as special value, since this is possibly the most widely performed activity in daily living and considered for recognition in most systems. All these results demonstrate the importance of not only seeking the best performance, but also considering an adequate windowing procedure.

## 5. Discussion

Although signal segmentation is a very important part of activity recognition systems, there is no clear consensus about how to apply it. Here, we provide an extensive study to bring light to this fact, a study that could be roughly summarized in two main conclusions: window size significantly matters, and short windows normally lead to better recognition performances. From the global analysis, the interval 1–2 s proves to provide the best trade-off between recognition speed and accuracy. The use of larger windows is seen to be required when simple feature sets are employed, while this turns out to not be necessary when richer feature sets are used, thus demonstrating the strong relation between the featuring and windowing processes.

The obtained results help reject the generalized idea of considering that the more data used for the feature extraction, the more accurate the recognizer is. Previous work demonstrated that long window sizes are normally required to capture the high motion variability found in activities with a complex description, such as household activities; however, many others may benefit from shorter window sizes. Activities that involve the complete body or several parts are more easily recognized and also permit one to optimize the window duration. Examples of these activities are walking, jogging or running, as well as other sports exercises. These activities are better described than those that only involve some body parts, as happens to occur for some sorts of jumps and some individual limb movements. In that case, some of the data windows captured from some body parts are not of much utility for discrimination, and the recognition process relies on a reduced set of informative windows. To compensate for this, further data are needed from the more informative ones (*i.e.*, larger data windows).

### Design guidelines

As is demonstrated in this study, in many cases, a subtle reduction in the system performance allow us to significantly shorten the window size. This is specially important for those applications that require a rapid detection, such as fall or epileptic seizure detectors. Moreover, other activities are better recognized for shorter window sizes. When designing an activity recognition system, the expert may need to prioritize detection performance or speed or even both. In most cases, a trade-off between both characteristics is required. One of the initial objectives of this work was to provide a reference tool to help designers to select an adequate segmentation configuration for the particular problem considered. In the following, specific windowing guidelines are provided for common activity categories based on the body parts they involve (legs, arms, back, waist and combinations), the intensity of the actions (energetic, non-energetic), mobility (translation) or their specific application domain (rehabilitation, military, gaming, sports and wellness).

The complete set of results and figures provided in Section 4 are here profited from to elaborate specific conclusions and guidelines devised to be generalized to other recognition systems and applications. Concretely, for each activity category, the minimum window sizes are provided that permit a reasonable (*F*_1_-*score* > 0.85) recognition performance (W*_min_*___*_size_,* recognition speed prioritization) and the window sizes that allow for optimal recognition capabilities (*W_max_*___*_perf_*, recognition performance prioritization). These window values are obtained through comparing the performance across all classification methodologies (DT, KNN, NB, NCC) for the most promising feature set (FS3) and for all the activities considered for each specific category. Through this, it is sought to achieve a generalization of the conclusions beyond the particular results obtained for each individual methodology.

Firstly, the activities are categorized based on the body parts that they principally involve during their execution. Correspondingly, activities in which arm movements are seen (*i.e.*, 19–25) may be reasonably recognized for a *W_min_*___*_size_* ranging between 0.5 and 1 s. An optimal recognition of these activities requires *W_max_*___*_perf_* values between 0.75 (shoulders low-amplitude rotation) and 2.25 s (frontal hand claps). Non-translation actions that predominantly involve the legs (*i.e.*, 26–30) require a *W_min_*___*_size_* that spans from 0.25 (rotation on the knees) to 1.75 (knees alternating to the breast), while an optimal recognition is achieved for *W_max_*___*_perf_* between 1.25 to 5.75 s. Activities that basically involve trunk movements (*i.e.*, 12, 15 and 17) require *W_min_*___*_size_* values between 0.5 to 1.25 s, while a maximal recognition is obtained for *W_max_*___*_perf_* 0.75 s for waist rotation, 2.25 s for repetitive forward stretching and 6.25 s for lateral bending. Other activities involve the motion of combinations of the former body parts. For example, exercises involving trunk and arm movements (*i.e.*, 9–11, 13 and 16) are optimally recognized for *W_max_*___*_perf_* in the range of 1–4.5 s, while the minimum window size is observed for *W_min_*___*_size_* values between 0.5 to 1.25 s. Movements of the trunk and legs are observed in actions (*i.e.*, 30 and 33) that may be maximally recognized for *W_max_*___*_perf_* values between 1–1.25 s and a *W_min_*___*_size_* of 0.25 s. Finally, activities that generally involve the movement of all the body parts (*i.e.*, 2–8, 18, 28, 29, 31 and 32) may be recognized for *W_min_*___*_size_* between 0.25 and 3.25 s and optimally identified for *W_max_*___*_perf_* that range from 0.5 s for rowing to 4 s for upper trunk and lower body opposite twist.

For those activities that determine an effective translation of the subject (*i.e.*, 1–3), it is seen that *W_min_*___*_size_* spans from 0.25 to 0.5 s, while a maximum performance is obtained for *W_max_*___*_perf_* values between one and 1.5 s. The window size requirements significantly vary among the five types of jumps analyzed in this work (*i.e.*, 4–8). A minimum window size of 0.5 s is possible for the detection of the activity, jumping, opening and closing legs and arms, whilst a *W_min_*___*_size_* of 3.25 s is required to identify the jumping front and back exercise. Minimum window sizes for the rest of the jumps are within an interval of 0.5 to 3.25 s. To achieve a maximum recognition performance, the window size must be enlarged. Thus, *W_max_perf_* spans from 1.75 s for the jumping sideways exercise to 6.75 s for jumping rope.

Another categorization may be performed considering the intensity of the activities, here defined as energetic and non-energetic activities. Energetic activities (*i.e.*, 1–8, 18, 23, 26, 28, 29, 31–33) can be reasonably recognized for *W_min_*___*_size_* values that range from 0.25 to 3.25 s, while for an optimal recognition, *W_max_*___*_perf_* should be between one and 3.25 s. The remaining activities (*i.e.*, 9–17, 19–22, 24, 25, 27, and 30), here classified as less- or non-energetic, are best recognized for *W_max_*___*_perf_* between 1.25 and 5.75 s, while the minimum window size *W_min_*___*_size_* ranges between 0.25 and 2 s.

The activities considered in this study could be seen as part of the target set of actions devised for some activity recognition applications. Here, various application domains are identified, taking into account the characteristics of these activities. The first domain corresponds to exercises for rehabilitation purposes. Activities involving legs, the trunk and legs and the complete body (*i.e.*, 2–8, 18, 26–33) could be part of the rehabilitation or stimulation exercises of the lower body. For these activities, a *W_max_*___*_perf_* ranging between 1.25 and 3.25 s is required for an optimal recognition, whilst the minimum window size spans from *W_min_*___*_size_* 0.25 to 3.25 s. Rehabilitation exercises for the upper body involve arms and the trunk and arm activities (*i.e.*, 9–11, 13, 16, 19–25) that are optimally recognized for *W_max_*___*_perf_* between 1.25 and 2.25 s, while *W_min_*___*_size_* values range from one to 1.25 s. Activities similar to the one considered in the training of security and military forces or bodies (*i.e.*, 1–6, 18, 26 and 27) require *W_min_*___*_size_* values between 0.25 and 2 s and *W_max_*___*_perf_* values between one and 5.75 s. Some of the analyzed activities could be also within the scope of specific gaming applications. For example, some games may require detecting some sort of jumps (*i.e.*, 4–8), hits (*i.e.*, 20, 23) or dance steps (*i.e.*, 12, 18, 19, 22, 28). The window size values presented above for the jump category may be likewise applied to games involving jumps. The recognition of hits require a *W_min_*___*_size_* of 1 s and *W_max_*___*_perf_* values between 1.25 and 1.75 s. For the movements identified to be usable in dance games, the *W_min_*___*_size_* may range between 0.5 and 2 s, whereas the *W_max_*___*_perf_* values span between 0.75 and 4 s. The last application domain corresponds to wellness and sports. The activities considered in this work may be part of warm up and cool down routines typically performed before sports practice, as well as fitness exercises normally performed during wellness training. For this case, all the activities may be considered. For an optimal recognition of the activities, *W_max_*___*_perf_* is seen to range between 0.5 and 6.75, while the *W_min_*___*_size_* spans from 0.25 to 3.25 s. All these guidelines are summarized in Table 5.

Clearly, the generalization of these results to other systems is not that simple, since each particular application may have specific requirements or the activities may be different to those considered here. Accordingly, the authors rather propose these guidelines as a hint to help orientate designers within the task of ascertaining which window size should be preferentially utilized. A good practice would consist in evaluating the recognition system capabilities for diverse window size values within the interval identified according to the recognition priority (speed or performance).

### Study generalization

For the sake of generalization, the tested recognition systems here correspond with the ones the most widely used in related works. Moreover, simplicity and comprehensiveness were key elements that were born in mind during the selection of the models, thereby allowing us to focus on the potential impact of the segmentation stage. Thus, for example, data directly captured through the sensors are used, avoiding any kind of filtering or preprocessing. These procedures normally remove some parts of the raw signals that may potentially lead to a change in the signal space, which may limit the applicability of these results to other designs. Moreover, the features used are very simple, easy to calculate and with interpretable physical meaning. Concretely, the “mean” allows us to extract the contribution to the acceleration from the gravitational component, which is particularly informative for distinguishing among sedentary or low-intensity activities. The “standard deviation”, “minimum” and “maximum” provide insights into the intensity and magnitude of the movements, while the “mean crossing rate” correlates with the dynamicity and frequency of the executions. Similar tendencies have been found for the various feature sets for each independent classification methodology, thus demonstrating that the results obtained here could be extrapolated to other systems of a similar nature. In either case, the differences among performance quality for each feature set determine that an automatic selection of better features could possibly lead to improved results. The generalization of the results and, principally, the provided guidelines is also achieved through the use of some of the most widely used standard classification methodologies in the activity recognition domain. This makes it possible to decouple the conclusions obtained for each activity category from each particular classification paradigm.

### Sampling rate

One may argue that the results presented in this study may be subject to the considered signal sampling rate. Although the amount of data that conforms a given window depends on this, we consciously decided to define the experiments in terms of time, since this is a magnitude common to any activity realization. Therefore, the results obtained here could be, in principle, applied to other monitoring systems with a different sampling rate.

### Performance metrics

The recognition capabilities of a given system are normally measured in terms of accuracy. Despite this metric having been and being extensively used in many fields, its use is only recommended for those problems in which there are no imbalance issues [84]. Because of this, in this work, we rather used the *F*_1_-*score* metric, which lacks this sort of limitation. Consequently, the results obtained in this work could be generalized for each activity independently of the number of available instances for each target activity.

### Challenges and limitations

The presented results have been provided just for acceleration data; however, current tendencies show that the use of other sensing modalities could help to improve recognition performance and system robustness. Gyroscopes and magnetometers are more and more frequently used in combination with accelerometers for recognition purposes. Although accelerometers have proven to suffice, an analysis with these other modalities could be of interest. Moreover, a similar study of this could be also valuable for other activity recognition domains, such as for computer vision or ambient intelligence.

One of the main conclusions derived from this work is that activities involving several body parts are more easily recognizable and allow for shorter window sizes. To monitor several body parts, a setup, such as the one considered in this work consisting of several sensors, is required. Therefore, the results presented here are of limited application to those systems that rely on a very reduced set of sensors or even a unique device. Nevertheless, the latest contributions show that ensuring robustness and guaranteeing a reasonable recognition rate demands a complete monitoring of the body as much as the number of target activities and their diversity increases [85,86]. Thereby, we consider that this study perfectly suits current and, especially, future trends.

## 6. Conclusions

The activity recognition process consists of several stages, each one of crucial importance. One of these steps is signal segmentation, which is normally performed through a windowing procedure. Despite the importance of selecting an appropriate window size, most designs rely on randomly selected values or figures used in previous cases of success, which nevertheless could not optimally apply to the particular considered problem. As a consequence, very limited knowledge and consensus exists in this respect.

In this work, we have presented an extensive study that analyzes the effects of the windowing process on activity recognition system performance. Several methodologies extensively used in previous works are used for evaluation. From the results, reduced windows (2 s or less) are demonstrated to provide the most accurate detection performance. In fact, the most precise recognizer is obtained for very short windows (0.25–0.5 s), leading to the perfect recognition of most activities. Contrary to what is often thought, this study demonstrates that large window sizes do not necessarily translate into a better recognition performance.

This work is found of utility not only for the sake of research, but for system design purposes. System configuration and design tasks may benefit from the figures provided as part of this work. A set of guidelines for the windowing process design has been particularly defined for different activity categories and applications. These guidelines are not seen to replace the need for the search of the optimal window size configuration during the design phase, but rather, provide a reference for the activity recognition system designer. The next steps include extending the scope of this study to other activity recognition domains and technologies.

## Figures and Tables

**Table 1.** Principal segmentation techniques.

Activity-defined windows	Sekine *et al.* (2000) [10], Lester *et al.* (2006) [13], Nyan *et al.* (2006) [11], He and Jin (2009) [15], Gu *et al.* (2009) [32], Gyorbiro *et al.* (2009) [33], Khan *et al.* (2010) [34], Hong *et al.* (2010) [35], Figo *et al.* (2010) [16], Dernbach *et al.* (2012) [14], Yoshizawa *et al.* (2013) [12]
Event-defined windows	Aminian *et al.* (1999) [17], Aminian *et al.* (2002) [19], Mansfield and Lyons (2003) [36], Zijlstra and Hof (2003) [37], Zijlstra (2004) [38], Selles *et al.* (2005) [18], Jasiewicz *et al.* (2006) [20], Ward *et al.* (2006) [39], Benocci *et al.* (2010) [21], Sant'Anna and Wickström (2010) [22], Dobkin *et al.* (2011) [24], Aung *et al.* (2013) [23]
Sliding windows	Mantyjarvi *et al.* (2001) [40], Kern *et al.* (2003) [41], Krause *et al.* (2003) [42], Bao and Intille (2004) [29], Huynh and Schiele (2005) [43], Ravi *et al.* (2005) [44], Maurer *et al.* (2006) [45], Parkka *et al.* (2006) [46], Pirttikangas *et al.* (2006) [25], Huynh *et al.* (2007) [47], Lovell *et al.* (2007) [48], Suutala *et al.* (2007) [49], Amft and Troster (2008) [50], Stikic *et al.* (2008) [27], Preece *et al.* (2009) [30], Altun and Barshan (2010) [51], Han *et al.* (2010) [52], Khan *et al.* (2010) [53], Marx (2010) [31], Sun *et al.* (2010) [54], Atallah *et al.* (2011) [55], Gjoreski and Gams (2011) [56], Jiang *et al.* (2011) [57], Kwapisz *et al.* (2011) [58], Lee and Cho (2011) [59], Siirtola and Röning (2012) [60], Wang *et al.* (2012) [61], Hemalatha and Vaidehi (2013) [62], Mannini *et al.* (2013) [26], Nam and Park (2013) [63], Nam and Park (2013) [64], Zheng *et al.* (2013) [65]

**Table 2.** Studies that use the sliding window approach (Part 1).

**Publication (Number of Subjects)**	**Activities (Number of Activities)**	**Accelerometer Placements (Number of Accelerometers)**	**Inter-Subject Classification Accuracy**	**Window Sizes (in seconds)**
Mantyjarvi *et al.* (2001) (1 subject) [40]	Level walking, stairs up/down, opening doors (4)	Left and right sides of the hip (2)	MLP (83%–90%)	2
Kern *et al.* (2003) (1 subject) [41]	Sitting, standing, shaking hands, writing on a keyboard and more (8)	Ankle, knee, hip, wrist, elbow, shoulder on both sides (12)	NB (∼90%)	∼0.5
Krause *et al.* (2003) (2 subjects) [42]	Walking, running, sitting, knee-bends, waving arms, climbing stairs and more (8)	Back of the upper arm (2)	K-means clustering, 1st order Markov	8
Bao and Intille (2004) (20 subjects) [29]	Walking, running, scrubbing, brushing teeth and more (20)	Upper arm, wrist, thigh, hip, ankle (5)	DT (84%) kNN (83%) NB (52%)	∼6.7
Huynh and Schiele (2005) (2 subjects) [43]	Walking, jogging, hopping, skipping and more	Shoulder strap (1)	NCC (∼80%)	0.25, 0.5, 1, 2, 4
Ravi *et al.* (2005) (2 subjects) [44]	Walking, running, standing, vacuuming and more (8)	Waist (pelvic region) (1)	NB (64%) SVM (63%) DT (57%) kNN (50%)	5.12
Maurer *et al.* (2006) (6 subjects) [45]	Walking, running, standing, sitting, upstairs, downstairs (6)	Wrist, belt, shirt pocket, trouser pocket, backpack, necklace (6)	DT (87%) kNN (<87%) NB (<87%)	0.5
Parkka *et al.* (2006) (16 subjects) [46]	Walking, running, rowing, Nordic walking and more (8)	Chest, wrist (2)	DT (86%) MLP (82%) Hierarchical (82%)	4, 10
Pirttikangas *et al.* (2006) (13 subjects) [25]	Walking, lying down, cycling, typing, vacuuming, drinking and more (17)	Right thigh and wrist, left wrist and necklace (4)	MLP (80%) kNN (90%)	0.1, 0.2, 0.5, 0.7, 1, 1.5
Huynh *et al.* (2007) (1 subject) [47]	High-level (going shopping, preparing for work, doing housework) (3) + Low-level (brushing teeth, taking a shower and more) (16)	Wrist, hip, thigh (3)	SVM (91.8%) kNN (83.4%) k-means (84.9%) HMMs (80.6%) for high-level SVM (79.1%) kNN (77%) k-means (69.4%) HMMs (67.4%) for low-level	6
Lovell *et al.* (2007) (52 subjects) [48]	Walking patterns (slope-down, slope-up, flat, stairs-down, stairs-up) (5)	Waist (1)	MLP-RFS (92%) MLP-RR (88.5%)	∼2.56
Suutala *et al.* (2007) (13 subjects) [49]	Lying down, vacuuming, typing, cycling, reading a newspaper, drinking and more (17)	Right thigh and wrist, left wrist, necklace (4)	17 activities (SVM (90.6%) HMM (84.2%) SVM-HMM (84.4%) DTS (93.6%))	0.7
			9 activities (SVM (94.1%) HMM (88.7%) SVM-HMM (90.4%) DTS (96.4%))	
Amft and Troster (2008) (6 subjects) [50]	Arm movements, chewing, swallowing (3)	Upper and lower arms (4)	Arm movements (79%) Chewing (86%) Swallowing (70%)	0.5
Stikic *et al.* (2008) (12 subjects) [27]	Housekeeping (vacuuming, sweeping, dusting, ironing, mopping and more) (10)	Wrist (1)	NB (57%) HMMs (60%) JB (68%)	0.5, 1, 2, 4, 8, 16, 32, 64, 128
Preece *et al.* (2009) (20 subjects) [30]	2 datasets: jogging, running, hopping, jumping and more (8) + Walking, climbing stairs up/down (3)	Waist, thigh, ankle (3)	kNN (96% with 8 activities; 98% with 3 activities)	2
Altun and Barshan (2010) (8 subjects) [51]	Sitting, playing basketball, standing, rowing, jumping and more (19)	Chest, both wrists and sides of the knees (5)	BDM (99.2%) LSM (89.6%) kNN (98.7%) DTW1 (83.2%) DTW2 (98.5%) SVM (98.8%) ANN (96.2%)	5
Han *et al.* (2010) (1 subject) [52]	Walking, running, standing, lying, falling, jumping (6)	Waist belt (1)	Fixed: HMM-P (78.8%) HMM-PNP (80.2%) Tilted: HMM-P (79.4%) HMM-PNP (53.2%)	0.32

**Table 3.** Studies that use the sliding window approach (Part 2).

**Publication (Number of Subjects)**	**Activities (Number of Activities)**	**Accelerometer Placements (Number of Accelerometers)**	**Inter-Subject Classification Accuracy**	**Window Sizes (in seconds)**
Khan *et al.* (2010) (6 subjects) [53]	Walking, upstairs, downstairs, running, sitting (5)	Smartphone in 5 different pocket locations (shirt's top, jeans' rear/front-left/front-right, coat's inner) (1)	ANN-OF (46%) ANN-LDA (60%) ANN-KDA (96%)	2
Marx (2010) (1 subject) [31]	Ball interactions (throwing, shaking, jerking sideways, holding very still) (4)	Embedded in iBall (1)	Heuristic (90%–95%)	0.666
Sun *et al.* (2010) (7 subjects) [54]	Walking, running, stationary, upstairs, downstairs, driving, bicycling (7)	Front/rear pockets on the trousers, front pockets on the coat (6)	SVM (93% with acceleration magnitude in 4 s; 92% without acceleration magnitude in 5 s)	1, 2, 3, 4, 5, 6
Atallah *et al.* (2011) (11 subjects) [55]	Reading, socializing, vacuuming and more (15)	Chest, arm, wrist, waist, knee, ankle, right ear (7)	kNN with k = 5 (∼56%) and k = 7 (∼64%), NB with Gaussian priors (∼61%)	5
Gjoreski and Gams (2011) (11 subjects) [56]	Standing, sitting, lying, sitting on the ground, on all fours, going down, standing up (7)	Chest, left thigh, right ankle (3)	Random Forest (93% only with chest; 96% adding left thigh; 98% with all accelerometers)	1
Jiang *et al.* (2011) (10 subjects) [57]	Walking, jogging, weight lifting, cycling, rowing and more (10)	Both forearms and shanks (4)	SVM ideal (95.1%) SVM with errors (75.2%) SVM without orientation errors (91.2%) SVM without errors (91.9%)	6.4
Kwapisz *et al.* (2011) (29 subjects) [58]	Walking, jogging, upstairs, downstairs and more (6)	Smartphone (1)	DT (85.1%) LR (78.1%) MLP (91.7%)	10
Lee and Cho (2011) (3 subjects) [59]	3 actions (walking, standing, climbing stairs) + 3 activities (shopping, moving by walk, taking bus)	Smartphone in the hand (1)	HHMM (84%) HMM (65%) ANN (65%)	5
Siirtola and Röning (2012) (8 subjects) [60]	Walking, running, cycling, sitting/standing, driving a car (5)	Smartphone in trousers' front pocket (1)	Offline (QDA (95.4%) kNN (94.5%)) Real-Time with Nokia (QDA (95.8%) kNN (93.9%)) Real-time with Samsung Galaxy (QDA (96.5%))	7.5
Wang *et al.* (2012) (8 subjects) [61]	Walking, jogging, upstairs, downstairs (4)	Smartphone (1)	GMM (91.2%) J48 (88.8%) LR (93.3%)	0.5, 0.8
Hemalatha and Vaidehi (2013) (5 subjects) [62]	Walking, sitting/standing, lying, falling (4)	Chest (1)	FBPAC (92%)	10
Mannini *et al.* (2013) (33 subjects) [26]	4 broad activity classes (ambulation, cycling, sedentary and other), daily activities (26)	Wrist or ankle (1)	SVM (84.7% with wrist, 95% with ankle) for 12.8 s	2, 4, 12.8
Nam and Park (2013) (3 subjects) [63]	Walking, toddling, crawling, wiggling, rolling and more (11)	Waist (1)	NB (81%) BN (87%) DT (75%) SVM (95%) kNN (96.2%) J48 (94.7%) MLP (96.3%) LR (93.2%)	∼2.7
Nam and Park (2013) (11 subjects) [64]	Walking, toddling, crawling, wiggling, rolling and more (10)	Waist (1)	NB (73%) BN (84.8%) DT (74%) SVM (86.2%) kNN (84.1%) J48 (88.3%) MLP (84.8%) LR (86.9%)	∼2.7
Zheng *et al.* (2013) (18/53/7 subjects) [65]	3 datasets: Walking, running, dancing and more (7) in 1*^st^* & 2*^nd^* / Walking, jogging, skipping and more (6) in 3*^rd^*	Wrist (1 in 1*^st^*) Hip (1 in 2*^nd^*) Waist pocket (1 in 3*^rd^*)	SWEM-SVM (94%/90%/82%) SVM (93%/89%/79%) ANN (91%/78%/74%)	10

**Table 4.** Warm up, cool down and fitness exercises considered for the activity set.

**Activity Set**		
L1: Walking	L12: Waist rotation	L23: Shoulders high-amplitude rotation
L2: Jogging	L13: Waist bends (reach foot with opposite hand)	L24: Shoulders low-amplitude rotation
L3: Running	L14: Reach heels backwards	L25: Arms inner rotation
L4: Jump up	L15: Lateral bend	L26: Knees (alternating) to the breast
L5: Jump front and back	L16: Lateral bend with arm up	L27: Heels (alternatively) to the backside
L6: Jump sideways	L17: Repetitive forward stretching	L28: Knees bending (crouching)
L7: Jump leg/arms open/closed	L18: Upper trunk and lower body opposite twist	L29: Knees (alternating) bending forward
L8: Jump rope	L19: Lateral elevation of arms	L30: Rotation on the knees
L9: Trunk twist (arms outstretched)	L20: Frontal elevation of arms	L31: Rowing
L10: Trunk twist (elbows bent)	L21: Frontal hand claps	L32: Elliptical bike
L11: Waist bends forward	L22: Frontal crossing of arms	L33: Cycling

**Table 5.** Summary of the windowing guidelines defined for diverse activity categories when prioritizing the recognition speed (*W_min_*___*_size_*) or the recognition performance (*W_max_*___*_perf_*).

**Category (Activities)**	***W****_min_*___*_size_***(s)**	***W****_max_*___*_perf_***(s)**
Arms (19–25)	0.5–1	0.75–2.25
Legs (26–30)	0.25–1.75	1.25–5.75
Trunk (12,15,17)	0.5–1.25	0.75–6.25
Trunk + arms (9–11,13,16)	0.5–1.25	1–4.5
Trunk + legs (30,33)	0.25	1–1.25
All body parts (2–8,18,28,29,31,32)	0.25–3.25	0.5–4
Translation (1–3)	0.25–0.5	1–1.5
Jumps (4–8)	0.5–3.25	1.75–6.75
Energetic (1–8,18,23,26,28,29,31–33)	0.25–3.25	1–3.25
Non-energetic (9–17,19–22,24,25,27,30)	0.25–2	1.25–5.75
Rehab lower body (2–8,18,26-33)	0.25–3.25	1.25–3.25
Rehab upper body (9–11,13,16,19–25)	1–1.25	1.25–2.25
Security/Military (1–6,18,26,27)	0.25–2	1–5.75
Gaming jumps (4–8)	0.5–3.25	1.75–6.75
Gaming hits (20,23)	1	1.25–1.75
Gaming dance (12,18,19,22,28)	0.5–2	0.75–4
Sport/Wellness (1–33)	0.25-3.25	0.5–6.75
